# Physiological and Functional Roles of Neurotrophin-4 During *In Vitro* Maturation of Porcine Cumulus–Oocyte Complexes

**DOI:** 10.3389/fcell.2022.908992

**Published:** 2022-07-08

**Authors:** Mirae Kim, Seon-Ung Hwang, Junchul David Yoon, Joohyeong Lee, Eunhye Kim, Lian Cai, Hyerin Choi, Dongjin Oh, Gabsang Lee, Sang-Hwan Hyun

**Affiliations:** ^1^ Veterinary Medical Center and College of Veterinary Medicine, Laboratory of Veterinary Embryology and Biotechnology (VETEMBIO), Chungbuk National University, Cheongju, South Korea; ^2^ Institute of Stem Cell & Regenerative Medicine (ISCRM), Chungbuk National University, Cheongju, South Korea; ^3^ Department of Biological Sciences, College of Arts and Sciences, University at Buffalo, The State University of New York (SUNY), Buffalo, NY, United States; ^4^ Division of Animal Sciences, University of Missouri, Columbia, MO, United States; ^5^ Laboratory of Molecular Diagnostics and Cell Biology, College of Veterinary Medicine, Gyeongsang National University, Jinju, South Korea; ^6^ Graduate School of Veterinary Biosecurity and Protection, Chungbuk National University, Cheongju, South Korea; ^7^ Department of Neurology, Institute for Cell Engineering, Johns Hopkins University School of Medicine, Baltimore, MD, United States

**Keywords:** neurotrophin-4, pig, cumulus cell, oocyte, *in vitro* maturation, *in vitro* fertilization, somatic cell nuclear transfer

## Abstract

Neurotrophin-4 (NT-4), a granulosa cell-derived factor and a member of the neurotrophin family, is known to promote follicular development and oocyte maturation in mammals. However, the physiological and functional roles of NT-4 in porcine ovarian development are not yet known. The aim of this study was to investigate the physiological role of NT-4-related signaling in the *in vitro* maturation (IVM) of porcine cumulus–oocyte complexes (COCs). The NT-4 protein and its receptors were detected in matured porcine COCs *via* immunofluorescence analysis. NT-4 was shown to promote the maturation of COCs by upregulating *NFKB1* transcription via the neurotrophin/p75^NTR^ signaling pathway. Notably, the mRNA expression levels of the oocyte-secreted factors *GDF9* and *BMP15*, sperm–oocyte interaction regulator *CD9*, and DNA methylase *DNMT3A* were significantly upregulated in NT-4-treated than in untreated porcine oocytes. Concurrently, there were no significant differences in the levels of total and phosphorylated epidermal growth factor receptor and p38 mitogen-activated protein kinase between NT-4-treated and untreated cumulus cells (CCs); however, the level of phosphorylated ERK1/2 was significantly higher in NT-4-treated CCs. Both total and phosphorylated ERK1/2 levels were significantly higher in NT-4-treated than in untreated oocytes. In addition, NT-4 improved subsequent embryonic development after *in vitro* fertilization and somatic cell nuclear transfer. Therefore, the physiological and functional roles of NT-4 in porcine ovarian development include the promotion of oocyte maturation, CC expansion, and ERK1/2 phosphorylation in porcine COCs during IVM.

## 1 Introduction

Interactions between oocytes and surrounding somatic cells in ovarian follicles, including granulosa and theca cells, are critical for follicular development in mammals (Matzuk et al., 2002). Among the somatic cells of ovarian follicles, mural granulosa cells produce and secrete autocrine/paracrine factors that coordinate follicular development (Canipari, 2000). Gonadotrophin-releasing hormone, epidermal growth factor (EGF), and an insulin-like growth factor are among many important intraovarian growth factors that are regulated through autocrine/paracrine mechanisms in the ovary. Many studies in the recent decades have demonstrated the essential role of the neurotrophin family, including neurotrophins and glial cell-derived neurotrophic factor, in mammalian ovarian development (Seifer et al., 2002a; Farhi et al., 2010; Chang et al., 2019). Neurotrophin-4 (NT-4), a granulosa cell-derived factor in the neurotrophin family, is known to promote follicular development and oocyte maturation in mammals (Seifer et al., 2002b; Van Den Hurk and Zhao, 2005). NT-4, which binds to the high-affinity tropomyosin-related kinase B (TrkB) receptor and low-affinity pan-neurotrophin receptor (p75^NTR^), is required for follicular assembly in humans and rodents (Harel et al., 2006; Childs et al., 2010). The NT-4/TrkB signaling pathway, in particular, is essential for oocyte survival and early follicular growth (Paredes et al., 2004).

As the ovary is a highly innervated organ and ovarian folliculogenesis is a highly regulated developmental process, various neurotrophic factors can facilitate the growth of nerve fibers and promote the development of follicular cells during ovarian development (Chang et al., 2019). Therefore, it is very important to investigate the interactions of neurotrophic factors with the female reproductive system. However, most studies have focused only on some of the former such as nerve growth factor (NGF), brain-derived neurotrophic factor (BDNF), and glial cell-derived neurotrophic factor (GDNF)–that are present in mammalian ovaries. Thus, more diverse and creative studies are needed to better understand the role of neurotrophic factors in female reproduction.

In mammalian cells, three major signaling pathways are activated by the tropomyosin-related kinase (Trk) receptor and its substrates: the mitogen-activated protein kinase (MAPK)/extracellular signal-regulated kinase (ERK) pathway (also known as the RAS/RAF/MEK/ERK pathway), phosphatidylinositol 3-kinase (PI3K) pathway, and phospholipase C-γ (PLC-γ) pathway (Reichardt, 2006). Among these, the activation of the MAPK/ERK signaling pathway in response to neurotrophins is critical for neuronal differentiation and survival (Jovanovic et al., 1996). The activation of the MAPK/ERK pathway is also involved in the regulation of oocyte microtubule organization, meiotic spindle assembly, and maintenance of metaphase II (MII) arrest (Fan and Sun, 2004). A previous study demonstrated that the supplementation of *in vitro* maturation (IVM) medium with BDNF improves the phosphorylation of TrkB and prolongs its activation time in mouse oocytes (Zhang et al., 2010). The authors speculated that the increase in MAPK phosphorylation in BDNF-treated mouse oocytes was not mediated by TrkB but was due to BDNF-induced phosphorylation of the cyclic AMP response element-binding protein (CREB) and to the activation of CREB-dependent transcription (Zhang et al., 2010). Another study demonstrated that BDNF accelerated the proliferation of Ishikawa human endometrial adenocarcinoma cells *via* the ERK1/2 signaling pathway (Cao et al., 2020). Several other studies have shown that BDNF/TrkB signaling pathway play an important role in follicular development (Dissen et al., 2009), granulosa cell proliferation (Dorfman et al., 2011), oocyte maturation (Seifer et al., 2002a), and embryonic development (Kawamura et al., 2005). There are many papers examining the physiological roles of BDNF in the female reproductive system, but little is known about the physiological roles of NT-4 and its associated signaling pathways in this system. In addition, the molecular mechanism by which NT-4 improves oocyte quality in porcine IVM medium is unknown.

Our previous study determined the optimal NT-4 concentration for porcine IVM and measured the relative expression levels of TrkB-related genes (Kim et al., 2021). In the present study, we investigated the physiological and functional roles of NT-4 in the maturation of porcine cumulus–oocyte complexes (COCs). NT-4 and its receptors were detected in matured cumulus cells (CCs) and oocytes by immunofluorescence staining. We also examined the relative mRNA expression levels of specific genes in NT-4-treated oocytes and investigated the expression levels of p38 MAPK and EGF receptor (EGFR) in the CCs and that of ERK1/2 in matured COCs using western blotting. Finally, subsequent developmental competence of embryos was assessed by analyzing embryonic cleavage (CL), blastocyst (BL) formation, and total cell counts after *in vitro* fertilization (IVF) and somatic cell nuclear transfer (SCNT).

## 2 Materials and Methods

### 2.1 Animal Treatment

Experimental procedures for obtaining pig fibroblasts were approved by the Committee on the Ethics of Animal Experiments of the Chungbuk National University (permission number: CBNUA-1415-20-02). The procedure to obtain ear tissues was conducted under anesthesia and efforts were made to minimize pain in the animals.

### 2.2 Chemicals and Reagents

Recombinant human NT-4 (450-04) was purchased from PeproTech (Rocky Hill, NJ, United States). It was dissolved in Dulbecco’s phosphate-buffered saline (DPBS; LB 001-02; WELGENE, Gyeongsan, Gyeongsangbuk-do, South Korea) containing 0.1% (w/v) bovine serum albumin (BSA). The 0 ng/ml group, which is the control group described in this text, refers to a group in which only DPBS containing 0.1% BSA as a vehicle was added without adding NT-4. All chemicals and reagents used in the present study were purchased from Sigma–Aldrich Corporation (St. Louis, MO, United States), unless otherwise indicated.

### 2.3 Oocyte Collection and IVM

Porcine ovaries were collected at a local slaughterhouse and transferred to the laboratory within 3 h in 0.9% (w/v) NaCl at 37°C–39°C. Oocyte collection and IVM were performed as previously described (Kim et al., 2021). Briefly, COCs were aspirated from 3–6-mm ovarian follicles using a 10-ml disposable syringe with an 18-G needle attached. After the follicular fluid was allowed to settle at 37°C for 5 min, the supernatant was removed and the precipitate was resuspended in HEPES-buffered Tyrode’s medium containing 0.05% (w/v) polyvinyl alcohol (TLH-PVA). The COCs with a homogeneous cytoplasm and more than three layers of compact CCs were selected under an SZX-ILLK100 stereomicroscope (Olympus Optical Co., Ltd., Tokyo, Japan). Approximately 60 COCs were transferred to each well of a 4-well dish (Nunc, Roskilde, Denmark) containing 500 μl of IVM medium (TCM199; Gibco, Grand Island, NY, United States) supplemented with 0.6 mM cysteine, 0.91 mM sodium pyruvate, 10 ng/ml EGF, 75 μg/ml kanamycin, 1 μg/ml insulin, and 0.1% (w/v) PVA. In the first 22 h of IVM, COCs were cultured in the maturation medium with 10 IU/ml equine chorionic gonadotropin and 10 IU/ml human chorionic gonadotropin, and in the next 20 h of IVM, COCs were cultured without equine and human chorionic gonadotropins. The concentration of NT-4 in the maturation medium was maintained at 0 or 10 ng/ml throughout the entire IVM period. All IVM procedures were performed in a humidified incubator (Astec, Fukuoka, Japan) at 39°C with 5% CO_2_.

### 2.4 Immunofluorescence Staining

After a total of 42 h of IVM, matured COCs were washed with TLH-PVA medium, denuded using 0.1% (w/v) hyaluronidase, and subjected to immunofluorescence staining. For fixation, permeabilization, and blocking, the Image-iT™ fixation/permeabilization kit (R37602; Invitrogen, Carlsbad, CA, United States) was used. Matured COCs were incubated with a fixative solution (4% formaldehyde) for 30 min at room temperature (RT). After fixation, the matured COCs were washed three times with DPBS with CaCl_2_ and MgCl_2_ (LB 001-01) containing 0.1% (w/v) PVA for 5 min each, then permeabilized for 30 min, and washed three times with DPBS containing 0.1% PVA. To eliminate a non-specific background, the Image-iT™ FX signal enhancer (Invitrogen) was used for 30 min, and then the matured COCs were washed three times again. After incubation for 1 h at RT with a blocking buffer (10% goat serum in DPBS), the matured COCs and oocytes were incubated with primary antibodies in the blocking buffer overnight at 4°C. The antibodies used in this experiment are listed in Supplementary Table S1. On the next day, the matured COCs were washed three times with DPBS for 10 min each on a shaker at 100 rpm and incubated with appropriate secondary antibodies at RT for 1 h. After the matured COCs were washed three times with DPBS for 10 min each on a shaker at 100 rpm, the nuclei were counterstained with 10 μg/ml Hoechst 33342. After mounting each slide using an antifade mounting solution (Molecular Probes, Inc., Eugene, OR, United States), the matured COCs were examined under a STELLARIS confocal laser microscope (Leica Microsystems, Bannockburn, IL, United States), and images were analyzed using the LAS X software.

### 2.5 Quantitative Reverse Transcription–Polymerase Chain Reaction

After IVM, MII oocytes and CCs were separately isolated and sampled from COCs using 0.1% (w/v) hyaluronidase. All samples were washed with DPBS and stored at−80°C until analysis. Total RNA was extracted using the TRIzol reagent (TaKaRa Bio, Inc., Otsu, Shiga, Japan), and cDNA was synthesized from the extracted RNA using a 5× reverse transcription master mixture (Elpis Bio, Inc., Chungcheongnam-do, Daejeon, South Korea) according to the manufacturer’s protocol. The synthesized cDNA (1.2 μg/μl for CCs and 0.5 μg/μl for oocytes) was mixed with 2× SYBR Premix Ex Taq (TaKaRa Bio, Inc.) and 10 pmol of specific primers (Macrogen, Inc., Seoul, South Korea) to perform quantitative real-time PCR (qPCR). The primers used in this study are listed in Supplementary Table S2. qPCR analysis was performed on a CFX96 Touch real-time PCR detection system (Bio-Rad, Hercules, CA, United States) using 40 cycles of denaturation at 95°C for 15 s, annealing at 57°C for 15 s, and extension at 72°C for 30 s. Fluorescence intensity was measured at the end of the extension phase of each cycle. All primers used in the experiments were designed using the Primer 3 software (ver. 4.0, http://bioinfo.ut.ee/primer3-0.4.0/). A relative standard curve approach was used to determine PCR efficiency of each primer (Pfaffl, 2001). The PCR efficiencies of each primer were found to be in an acceptable range (90%–110%). The cycle threshold was defined as the cycle number at which the amplified PCR product entered the exponential phase. The relative mRNA expression (R) was calculated using the equation R = 2^−[ΔCt sample–ΔCt control]^ (Schmittgen and Livak, 2008). The expression level of each mRNA was normalized to that of glyceraldehyde 3-phosphate dehydrogenase (*GAPDH*) for CCs and 18S ribosomal RNA (*RN18S*) for oocytes. These experiments were repeated at least three times.

### 2.6 Western Blotting

Western blotting was performed to analyze the protein expression and activation levels using previously described methods (Yoon et al., 2019a). After IVM, CCs were separately isolated and sampled from approximately 120–180 COCs using 0.1% (w/v) hyaluronidase. Total protein was extracted from the CCs using ProEX CETi lysis buffer (TransLab, Daejeon, Chungcheongnam-do, South Korea). The protein concentrations were measured using the Pierce™ bicinchoninic acid protein assay kit (Thermo Fisher Scientific, Waltham, MA, United States) with BSA as a standard, and the samples were stored at −80°C until analysis. Total proteins (15 μg per lane) were separated by 10% sodium dodecyl sulphate–polyacrylamide gel electrophoresis and then transferred onto a polyvinylidene fluoride membrane (Merck Millipore, Burlington, MA, United States) using a Mini-PROTEAN Tetra cell (Bio-Rad) according to the manufacturer’s instructions. The membranes were washed twice with Tris-buffered saline–Tween 20 (TBS-T) buffer (0.2 μM Tris, 1.37 M NaCl, and 0.05% Tween 20) for 10 min each and blocked for 5 min at RT with EveryBlot blocking buffer (Bio-Rad). The membranes were incubated with primary antibodies in EveryBlot blocking buffer (Bio-Rad) overnight at 4°C. On the following day, the membranes were washed three times with TBS-T buffer for 10 min each on a shaker at 100 rpm and then incubated with appropriate horseradish peroxidase-conjugated secondary antibodies at RT for 1.5 h. The antibodies used for western blotting are listed in Supplementary Table S3. After washing the membranes three times with TBS-T buffer, the target proteins were visualized using a chemiluminescent substrate kit (4:1 mixture of the SuperSignal West Pico PLUS chemiluminescent substrate and SuperSignal West Femto maximum sensitivity substrate; Thermo Fisher Scientific). Immunoreactive bands were detected using a Lumino Graph II imaging system (ATTO Corporation, Tokyo, Japan), and optical densities of target proteins were analyzed using the CS Analyzer 4 software. The densities of the bands were normalized to that of GAPDH, which was used as the loading control. The experiments were performed at least three times.

### 2.7 Capillary Western Blotting

Capillary western blot analyses were performed for MII oocytes using a chemiluminescent and fluorescent western blotting Jess system (ProteinSimple, Inc., Santa Clara, CA, United States) according to the manufacturer’s protocol (Rajput et al., 2021). After IVM, MII oocytes were isolated from each group and sampled from approximately 120–180 COCs as described in Section 2.6 using the same procedure for extracting and quantifying total protein. The protein samples (3 μg/μl) were separated using capillary cartridges (12–230 kDa Jess separation module; ProteinSimple, Inc.). Each sample was diluted with 0.1× sample buffer, mixed (4:1) with 5× fluorescent master mix (containing 5× sample buffer, 5× fluorescent standard, and 200 mM dithiothreitol), and heated at 95°C for 5 min to denature proteins. After cooling at 4°C, the denatured protein samples, blocking buffer, primary antibodies, horseradish peroxidase-conjugated secondary antibodies, and a chemiluminescent substrate (1:1 luminol/peroxidase mixture) were dispensed into designated wells in an assay plate. The antibodies used for capillary western blotting are listed in Supplementary Table S4. The electrophoretic separation and immunodetection steps were performed in a fully automated capillary system, and the relative expression levels of proteins were automatically calculated by the Compass for Simple Western software version 6.0 (ProteinSimple, Inc.). The experiments were performed at least three times.

### 2.8 IVF and *In Vitro* Culture (IVC)

IVF was performed as previously described (Yoon et al., 2019b). Fresh boar liquid semen was supplied twice a week by a local artificial insemination center (Xperm-Ⅴ; Darby Genetics, Inc., Anseong, South Korea) and stored at 17°C until use. Selected MII oocytes from matured COCs were washed twice with modified Tris-buffered medium (mTBM) (Abeydeera and Day, 1997), then transferred to 40-μl droplets (15 oocytes/drop) of mTBM, and incubated at 39°C in a 5% CO_2_ humidified incubator until fertilization. The liquid semen was washed twice with DPBS containing 0.1% BSA by centrifugation at 2,000 rpm for 2 min. Thereafter, the sperm pellet was resuspended in mTBM, which was pre-equilibrated overnight at 39°C and 5% CO_2_. The sperm motility was assessed under a stereomicroscope (Olympus), and only sperm with motility of greater than 70% were used for IVF. The sperm concentration was determined using a hemocytometer, and the sperm was appropriately diluted with mTBM. The MII oocytes were coincubated with the sperm at a final concentration of 5 × 10^5^ sperm/ml for 20 min at 39°C in a 5% CO_2_ humidified incubator (Astec). Thereafter, loosely attached sperm were removed from the zona pellucida (ZP) by gentle pipetting. The oocytes were washed twice and incubated in mTBM without sperm for 5–6 h at 39°C in a 5% CO_2_ humidified incubator (Astec). The presumptive IVF zygotes were washed and cultured in 25-μl droplets (10 embryos/drop) of porcine zygote medium 3 (PZM3) (Yoshioka et al., 2002) at 39°C for 168 h in a humidified incubator with 5% CO_2_/O_2_ and 90% N_2_. For IVC, the medium was changed to fresh 30-μl droplets of PZM3 at 48 and 96 h after IVF. On day 4, 10% fetal bovine serum (FBS) was added to the PZM3 droplets with embryos, and all droplets were covered with mineral oil (BP26291; Thermo Fisher Scientific).

### 2.9 Evaluation of Fertilization Parameters

To analyze the fertilization efficiency, zona-free IVF embryos were stained to detect sperm penetration into the oocyte, and pronucleus formation was assessed according to previous studies (Koo et al., 2005; Yoon et al., 2017). Briefly, 12 h after insemination, the ZP of presumptive IVF zygotes was removed using a prewarmed acidic Tyrode’s solution. The zona-free zygotes were washed three times with TLH-PVA and they were fixed with 3.7% paraformaldehyde in DPBS containing 0.1% PVA for 5 min at RT. The fixed embryos were then stained with 10 μg/ml Hoechst 33342 for 5 min and mounted on glass slides in 100% glycerol droplets. The number of penetrated spermatozoa, the sperm ratio, and the formation of the male pronucleus were investigated using a TE300 fluorescence microscope (Nikon, Tokyo, Japan) with an ultraviolet filter (370 nm). The parameters used to evaluate the fertilization efficiency are described below (Biswas and Hyun, 2011): Penetration rate refers to the percentage of oocytes penetrated by one or more sperm. Male pronucleus (MPN) formation rate means the percentage of oocytes with male pronuclei. Monospermy rate is defined as the percentage of penetrated oocytes with two pronuclei or one pronucleus together with one decondensed sperm head. Polyspermy rate is defined as the percentage of penetrated oocytes with multiple pronuclei. Efficiency of fertilization was assessed by measuring the percentage of monospermic oocytes from total examined oocytes.

### 2.10 Donor Cell Preparation

Pig fibroblasts were isolated from ears of Yucatan miniature pigs. Using scissors and forceps, hair and soft tissues present in the pig ear tissue were removed and discarded. Following washing three times with DPBS, the tissue was chopped with a surgical blade on a 100-mm Petri dish (SPL Life Sciences Co.,, Ltd., Pocheon-si, Gyeonggi-do, South Korea). The minced ear tissues were dissociated in 0.25% trypsin–EDTA (Gibco) for 1–2 h. The trypsinized cells were inactivated with a medium containing 10% (v/v) FBS and washed by centrifugation at 3,000 rpm for 2 min at least three times. Subsequently, the cells were seeded and cultured on a 100-mm culture dish (SPL Life Sciences Co., Ltd.) for 3–4 days in Dulbecco’s modified Eagle’s medium (high glucose) supplemented with 10% (v/v) FBS, 1× minimum essential medium nonessential amino acids, 1× GlutaMAX, 0.1 mM β-mercaptoethanol, and 1× antibiotic–antimycotic solution (all from Gibco) at 37°C in a humidified incubator with 95% air and 5% CO_2_. After removing unattached tissues, attached cells were further cultured until reaching confluency. The culture medium was changed every 2 days, and the fibroblasts were subcultured every 4–5 days. Porcine fibroblasts were cultured until they reached 90% confluency, then dissociated with 0.5% trypsin–EDTA (Gibco) for 1 min, and prepared for use in SCNT experiments.

### 2.11 SCNT and IVC

SCNT was performed according to Hwang et al. (2018a). After 42 h of IVM, denuded MII oocytes were chosen for enucleation. The selected MII oocytes were washed three times with calcium-free TLH medium containing 0.2% BSA and 5 μg/ml cytochalasin B. Enucleation was conducted using a micromanipulator with a 16-mm glass pipette (Humagen, Charlottesville, VA, United States). Thereafter, the trypsinized donor cells were transferred into the perivitelline space of enucleated oocytes. They were fused by two pulses of a 180 V/mm direct current for 60 μs in a 260 mM mannitol solution, which contained 0.001 mM CaCl_2_ and 0.05 mM MgCl_2_, using an LF201 electrical pulsing machine (Nepa Gene, Chiba, Japan). After electrical cell fusion, the SCNT embryos were incubated in 6-dimethyl aminopurine with 5 μg/ml cytochalasin B in 30-μl droplets (10 embryos/drop) of PZM3 for 4 h post activation. The SCNT embryos were washed and transferred to fresh PZM3 droplets for IVC at 39°C for 168 h in a humidified incubator with 5% CO_2_/O_2_ and 90% N_2_. The IVC protocol for SCNT embryos was identical to that previously described for IVF embryos (Section 2.8).

### 2.12 Assessment of Embryo Quality and Total Cell Counts

Day 0 was regarded as the day on which IVF or SCNT was initiated. On day 2, the CL rates of embryos were evaluated to assess their developmental competence, and the embryos were transferred to fresh PZM3 droplets. The normally cleaved embryos were classified into three groups, 2–3, 4–6, and 6–8 cells, while one-cell and fragmented embryos were excluded. On day 4, 10% FBS was added to the PZM3 droplets containing embryos. Finally, on day 7, the BLs formed were categorized into three groups as follows: early, expanded, and hatched BLs. All BLs from each group were collected, and the ZP was removed (except for hatched BLs) with 0.5% protease. Thereafter, the zona-free BLs were fixed with 3.7% paraformaldehyde in DPBS containing 0.1% PVA for 5 min and washed with DPBS containing 0.1% PVA. The fixed BLs were stained with 10 μg/ml Hoechst 33342 for 5 min, then mounted on glass slides in 100% glycerol droplets, and gently covered with coverslips. The slides were observed under a TE300 epifluorescence microscope (Nikon) with an ultraviolet filter (370 nm).

### 2.13 Statistical Analysis

All experiments were performed at least three times. All statistical data were analyzed using GraphPad Prism version 7.0 (GraphPad Software, Inc., San Diego, CA, United States). Statistical analysis was performed using an unpaired two-tailed Student’s t-test. All data are presented as the mean ± SEM. A value of *p* < 0.05 was considered to indicate statistically significant differences.

## 3 Results

### 3.1 Detection and Localization of NT-4 and Its Receptors in *In Vitro* Matured Porcine Oocytes and Cumulus Cells

**FIGURE 1:**
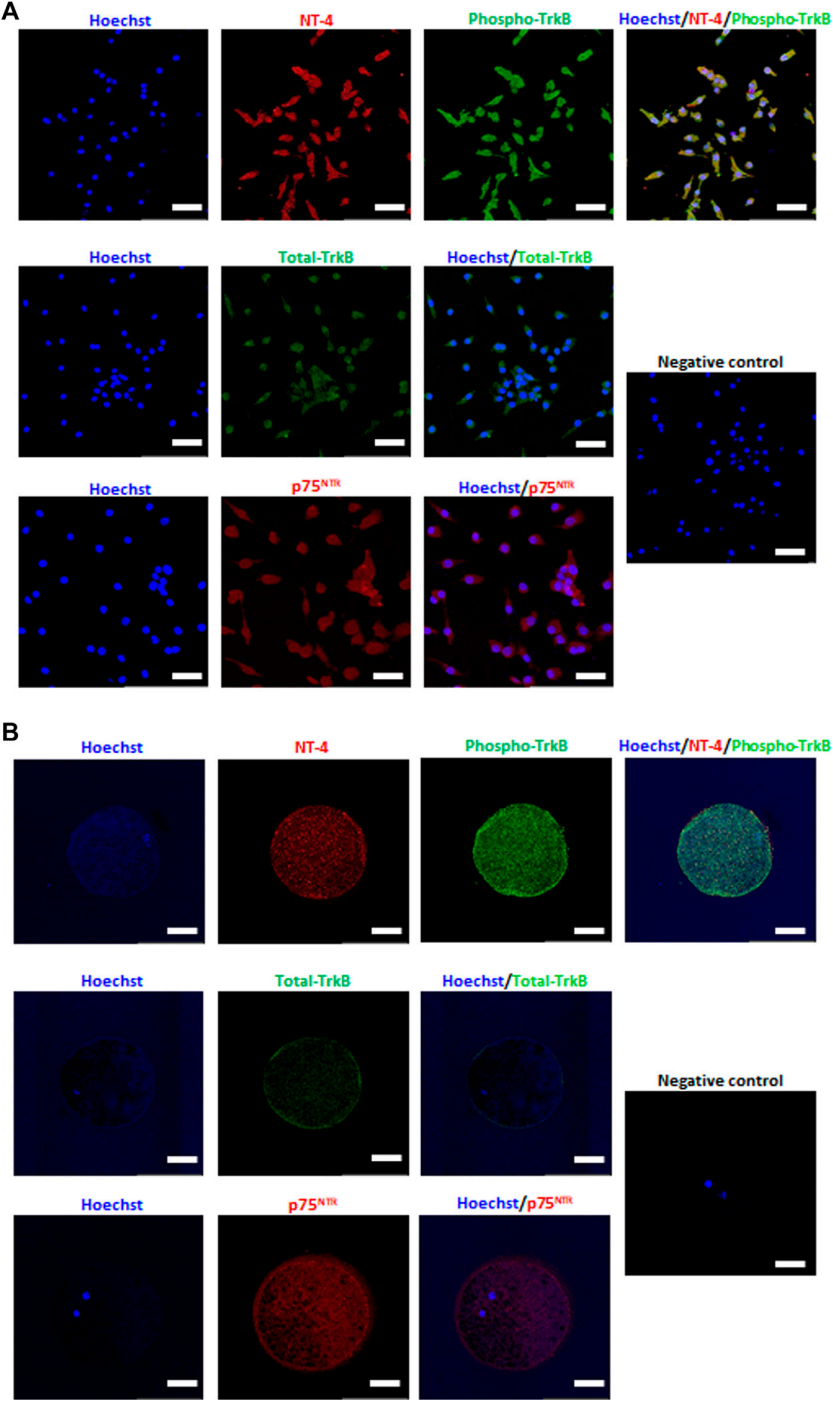
Detection and localization of neurotrophin-4 (NT-4) and its receptors in *in vitro* matured porcine cumulus cells (CCs) **(A)** and oocytes **(B)** by immunofluorescence analysis. Scale bars = 100 μm.

NT-4 and its related receptors were detected in *in vitro* matured porcine oocytes and CCs by immunofluorescence staining. In the CCs, high levels of NT-4 and phospho-TrkB were observed, and both total TrkB and p75^NTR^ were generally expressed (Figure 1A). Interestingly, in matured oocytes, NT-4 and phospho-TrkB were abundantly detected in the ooplasm; while total TrkB was mainly localized on the surface of the ooplasm, p75^NTR^ was evenly distributed in the ooplasm (Figure 1B).

### 3.2 Effects of NT-4 Supplementation During IVM on p75^NTR^-Related Gene Expression in Matured Porcine Oocytes and Cumulus Cells

**FIGURE 2:**
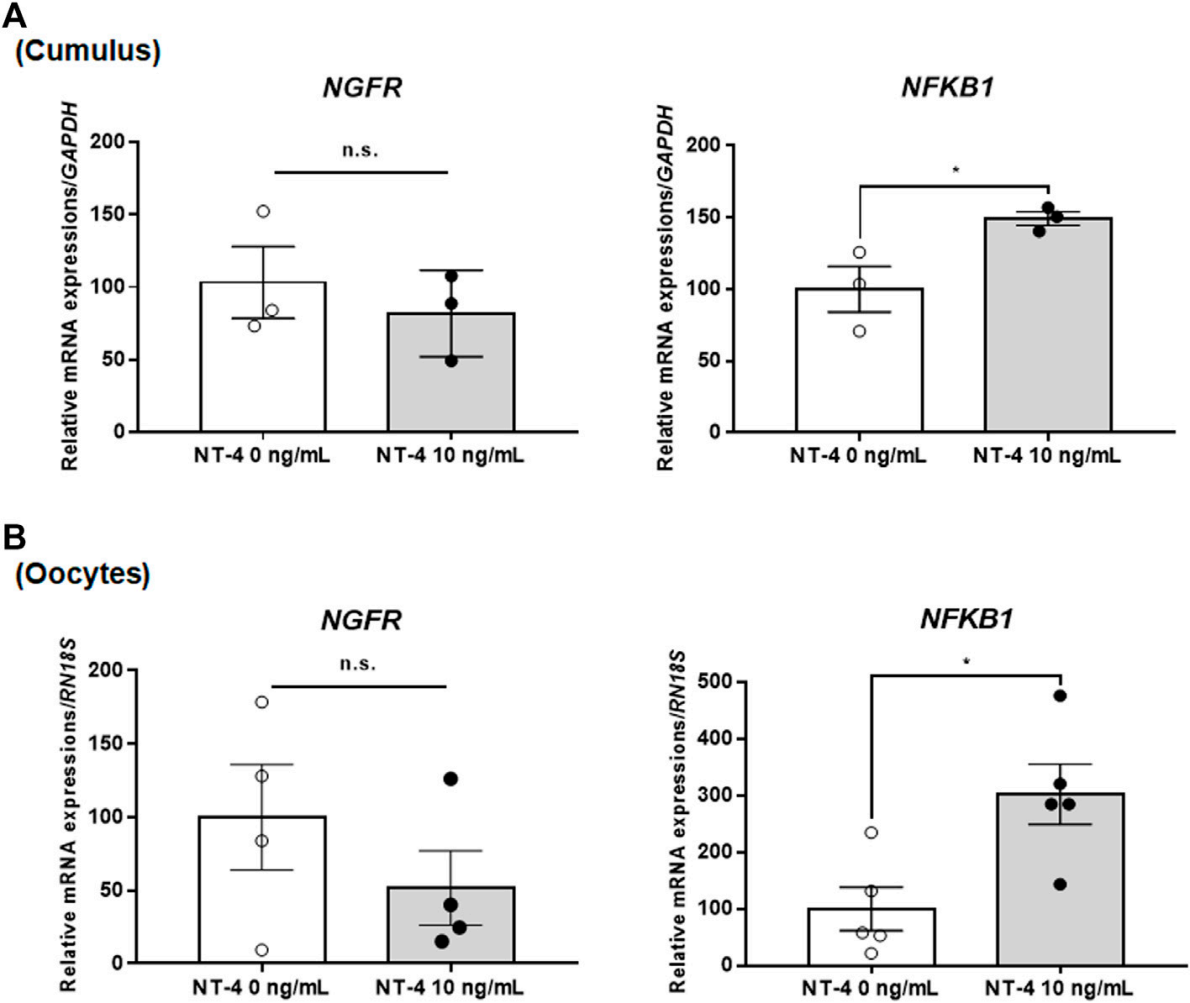
Relative mRNA expression levels of low-affinity pan-neurotrophin receptor (p75^NTR^)-related genes in neurotrophin-4 (NT-4)-treated porcine cumulus cells (CCs) **(A)** and oocytes **(B)**. Values represent the mean ± SEM. *p < 0.05 (Student’s t-test). The experiment was replicated at least three times. n.s. indicates not significant (p > 0.05).

Next, we analyzed the relative expression levels of p75^NTR^-related genes in NT-4-treated COCs during IVM. The mRNA expression levels of the *NGFR* gene (encoding p75^NTR^) in both NT-4-treated CCs and oocytes were not significantly different from that in the control. However, the mRNA expression levels of *NFKB1*, a downstream gene of *NGFR*, were significantly higher (*p* < 0.05) in both NT-4-treated CCs and oocytes than that in the control (Figure 2).

### 3.3 Effects of NT-4 Supplementation During IVM on Expression of Specific Genes in Matured Porcine Oocytes

**FIGURE 3:**
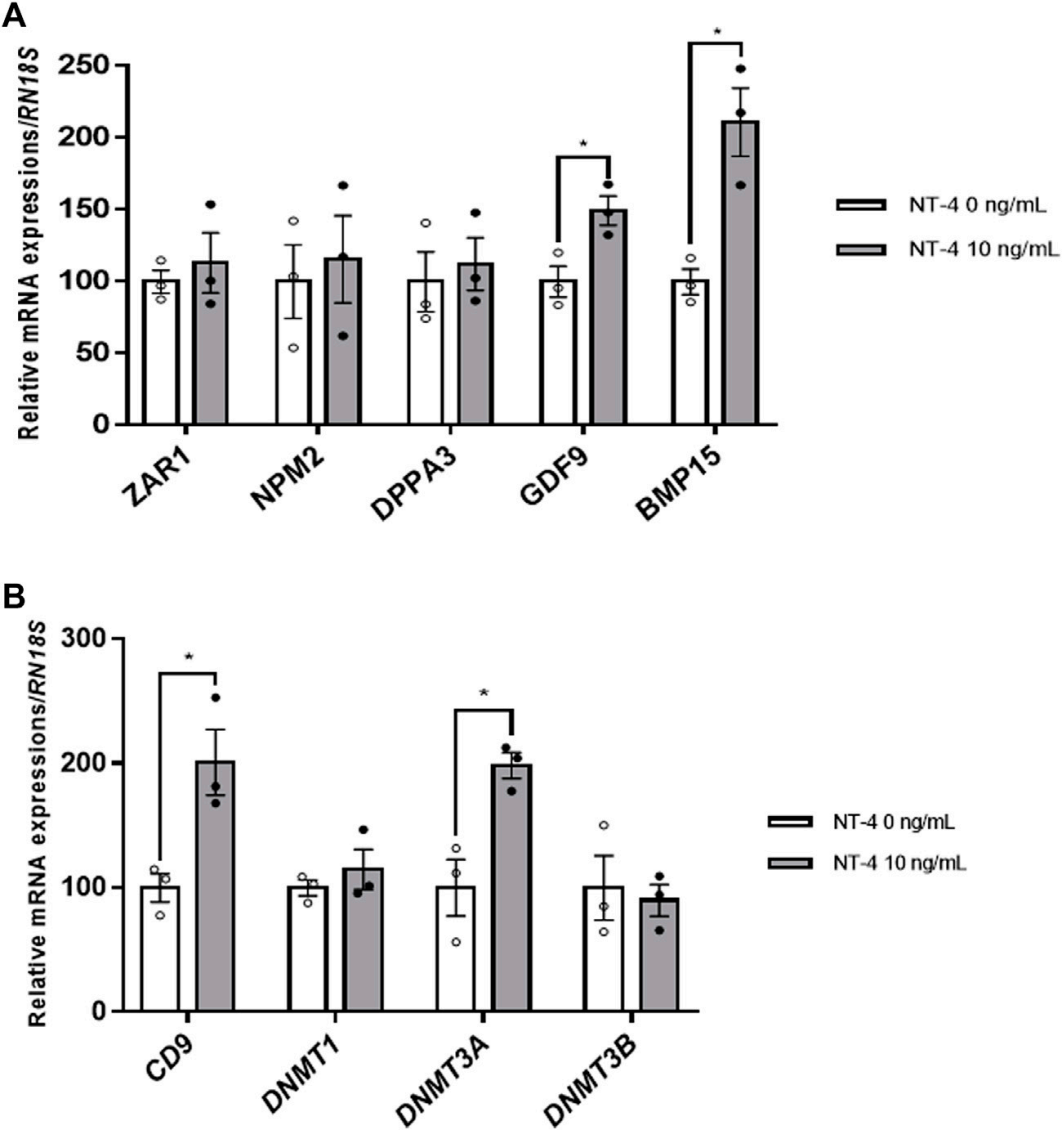
Relative mRNA expression levels of maternal factors, the regulator of sperm–oocyte interactions, and DNA methylation-related genes in neurotrophin-4 (NT-4)-treated porcine oocytes. **(A)** mRNA expression levels of maternal factor-related genes (*ZAR1*, *NPM2*, *DPPA3*, *GDF9*, and *BMP15*) **(B)** mRNA expression levels of the regulator of sperm–oocyte interactions (*CD9*) and DNA methylation-related genes (*DNMT1*, *DNMT3A*, and *DNMT3B*). Values represent the mean ± SEM. *p < 0.05 (*t*-test). The experiment was replicated at least three times.

Further, the effects of NT-4 treatment during IVM were examined on the expression levels of maternal factors, such as *ZAR1*, *NPM2*, *DPPA3*, growth differentiation factor 9 (*GDF9*), and bone morphogenetic protein 15 (*BMP15*), the sperm–oocyte interaction regulator *CD9*, and DNA methylation-related genes (*DNMT1*, *DNMT3A*, and *DNMT3B*) in porcine oocytes. Among the maternal factor-related genes, the mRNA expression levels of *GDF9* and *BMP15*, also called oocyte-secreted factors, were significantly higher (*p* < 0.05) in NT-4-treated oocytes than in the control (Figure 3A). Furthermore, the sperm–oocyte interaction regulator *CD9* showed a significantly higher (*p* < 0.05) transcription level in NT-4-treated oocytes than in the control (Figure 3B). In addition, porcine oocytes treated with NT-4 during IVM expressed a significantly higher (*p* < 0.05) *DNMT3A* level than that in the control (Figure 3B).

### 3.4 Underlying Mechanism of NT-4 Effects During Porcine IVM

**FIGURE 4:**
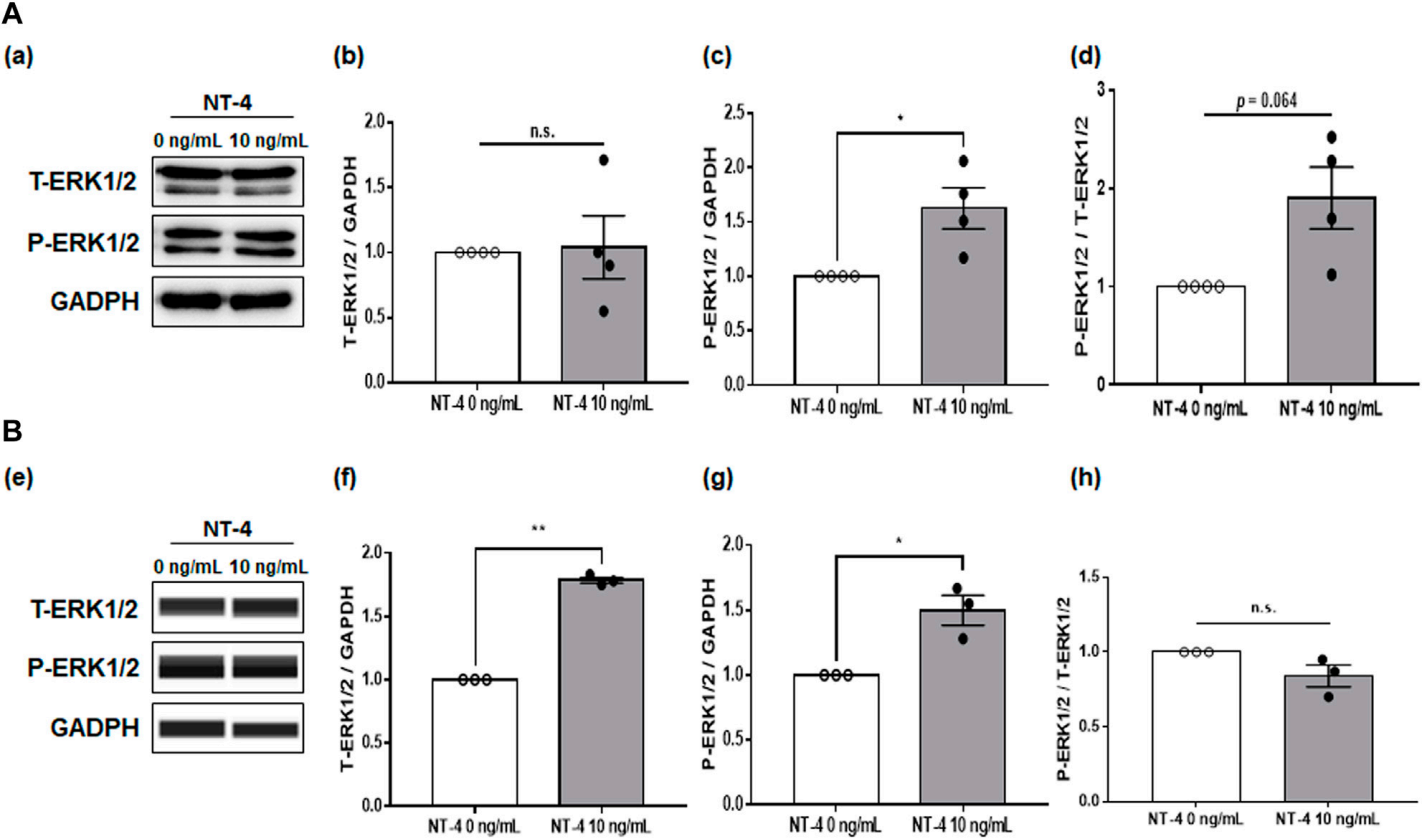
Expression and phosphorylation levels of extracellular signal-regulated kinase 1/2 (ERK1/2) in porcine cumulus cells (CCs) and oocytes after *in vitro* maturation (IVM). **(A,B)** Western blots of total ERK1/2 (T-ERK1/2), phosphorylated ERK1/2 (P-ERK1/2), and GAPDH in CCs **(A)** and oocytes **(B)**. The levels of T-ERK1/2 **(b, f)** and P-ERK1/2 **(c, g)** and the ratio of P-ERK1/2 to T-ERK1/2 **(d, h)** in CCs **(a)** and oocytes **(e)**. The results were normalized to those of GAPDH. Values represent the mean ± SEM. The experiment was replicated at least three times. *p < 0.05 and **p < 0.01 (Student’s t-test). n.s. indicates not significant (p > 0.05).

To investigate the physiological function of NT-4 during IVM of porcine COCs, the expression levels of the EGFR, p38 MAPK, and ERK1/2 proteins were first examined in NT-4-treated matured CCs. The results showed no significant differences in the levels of total EGFR and phosphorylated EGFR in NT-4-supplemented CCs (Supplementary Figure S1). The expression level of p38 MAPK, a regulator of follicular development in the CCs, was not significantly different in NT-4-treated CCs either (Supplementary Figure S2). Meanwhile, the level of phosphorylated ERK1/2 was significantly higher (*p* < 0.05) in NT-4-supplemented CCs than in the control (Figure 4A). Although, CCs that were treated with NT-4 during IVM showed a tendency for a higher ratio of phosphorylated ERK1/2 to total ERK1/2, the difference with the control value was not significant (*p* > 0.05). In the case of matured oocytes, the levels of total (*p* < 0.01) and phosphorylated ERK1/2 (*p* < 0.05) were significantly increased by NT-4 supplementation compared with those in the control (Figure 4B).

### 3.5 Effects of NT-4 Supplementation During Porcine IVM on Embryonic Developmental Competence After IVF and SCNT

**FIGURE 5:**
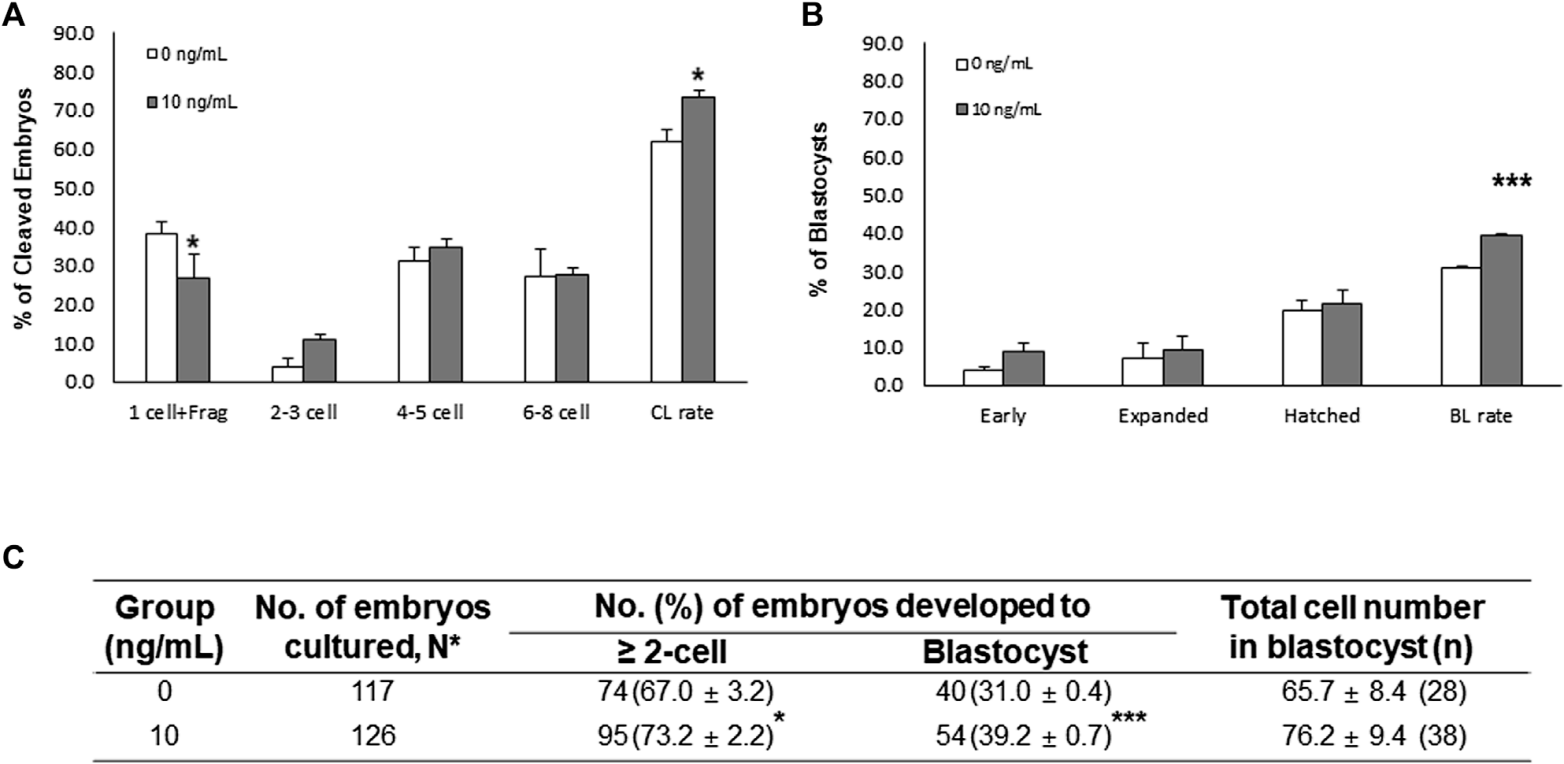
Effects of neurotrophin-4 (NT-4) treatment during *in vitro* maturation (IVM) on embryonic development after *in vitro* fertilization (IVF). **(A)** cleavage (CL) pattern and **(B)** blastocyst (BL) formation pattern of IVF embryos. *p < 0.05 and ***p < 0.001 (Student’s t-test). The CL rate was measured on day 2, and the BL formation rate was evaluated on day 7 of culture. Data are presented as the mean ± SEM from three replicate experiments **(C)** Summary of embryonic development after IVF.

To analyze the fertilization efficiency and developmental capacity in IVF experiments, MII oocytes were selected and coincubated with the sperm (5 × 10^5^ sperm/ml). Interestingly, the monospermy rate and fertilization efficiency were significantly (*p* < 0.05) higher in the NT-4-treated group than in the control group (Table 1). The CL and BL formation rates of IVF embryos were evaluated 2 and 7 days, respectively, after fertilization (Figure 5). When comparing the CL patterns on day 2, the percentage of one-cell plus fragmented embryos was significantly (*p* < 0.05) lower in the NT-4-treated group than in the control. The total CL rate after IVF was significantly higher (*p* < 0.05) in the NT-4-treated group than in the control (Figure 5A). On day 7, the total BL formation rate was significantly (*p* < 0.001) higher in the NT-4 treatment group than in the control (Figures 5B,C).

**TABLE 1:** Effect of neurotrophin-4 (NT-4) on sperm penetration of porcine oocytes during *in vitro* maturation (IVM) at 12 h post insemination.

Parameter	NT-4	
	0 ng/ml	10 ng/ml
No. of oocytes	34	40
Penetration (%)^a^	31 (92.5 ± 4.8)	39 (97.5 ± 2.5)
MPN Form (%)^b^	29 (94.1 ± 3.4)	34 (87.2 ± 4.7)
Monospermy (%)^b^	7 (21.9 ± 4.6)	17 (43.3 ± 7.1)*
Polyspermy (%)^b^	22 (72.2 ± 7.8)	17 (43.9 ± 11.5)
Efficiency of fertilization^c^	20.4 ± 4.4	42.5 ± 7.5*

a Percentage of the number of oocytes examined.

b Percentage of the number of oocytes penetrated.

c Efficiency of fertilization as the percentage of monospermic oocytes from total examined.

The data are given as the means ± SEM, from four replicate experiments. Data were analyzed by Student’s t-tests.

Asterisks indicate statistical significance (*p < 0.05) different. MPN, male pronucleus.

**FIGURE 6:**
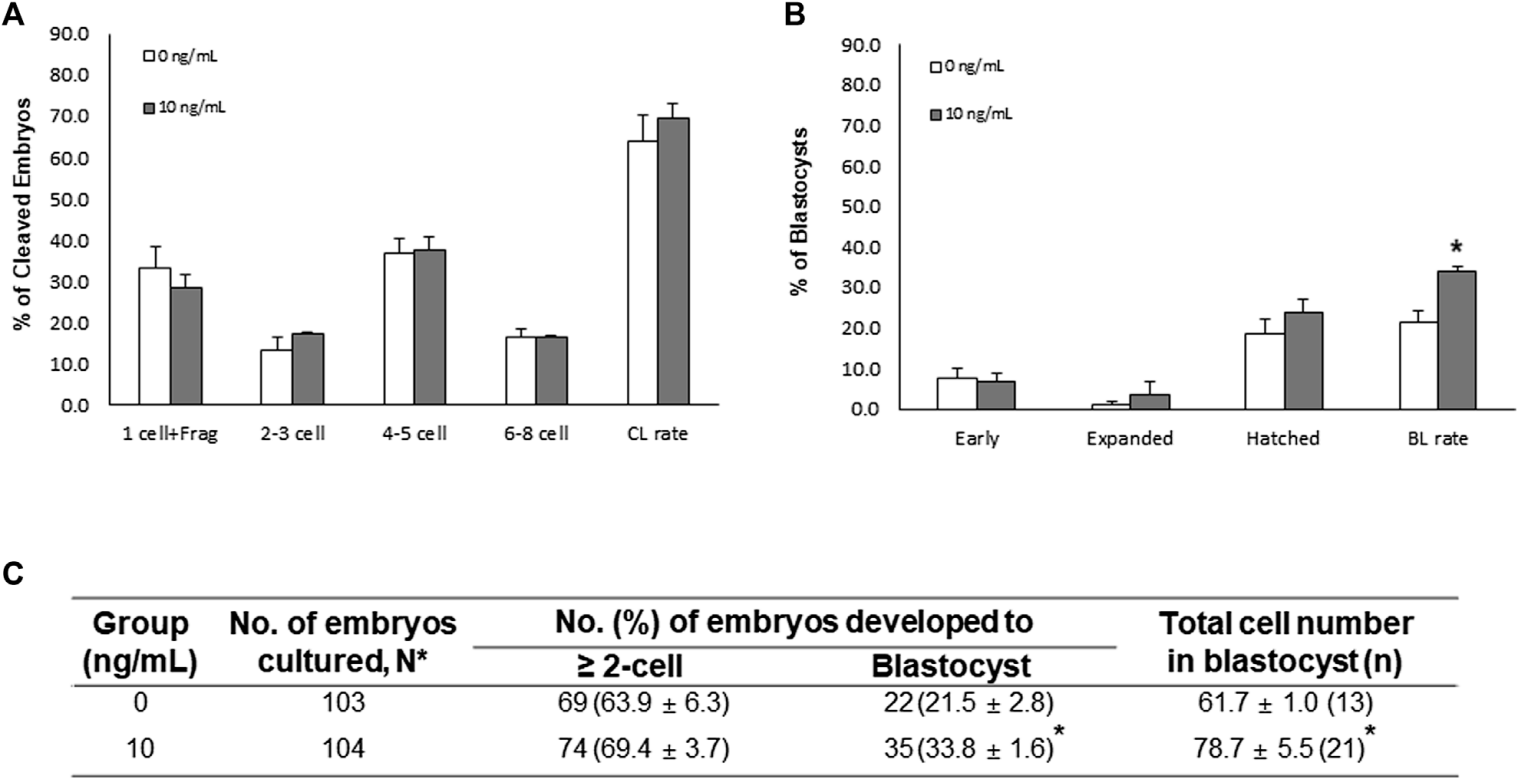
Effects of neurotrophin-4 (NT-4) treatment during *in vitro* maturation (IVM) on embryonic development after somatic cell nuclear transfer (SCNT). **(A)** cleavage (CL) pattern and **(B)** blastocyst (BL) formation pattern of SCNT embryos. *p < 0.05 (Student’s t-test). The CL rate was measured on day 2, and the BL formation rate was evaluated on day 7 of culture. Data are presented as the means ± SEM from three replicate experiments. **(C)** Summary of embryonic development after SCNT.

In the SCNT experiment, there were no significant differences in the CL pattern and CL rate of SCNT embryos between the control and NT-4 treatment groups (Figure 6A). However, on day 7, the BL formation rate and total cell number in BLs were significantly (*p* < 0.05) higher in the NT-4 treatment group than in the control (Figures 6B,C).

## 4 Discussion

Neurotrophic factors are very important in the development of the female reproductive system (Dissen et al., 2002). It has been demonstrated that NGF, BDNF, and GDNF enhance oocyte maturation and early embryonic development (Linher-Melville and Li, 2013). NGF plays an essential role in the female and male reproductive systems, and is known to promote ovulation while present in high concentrations in the semen of most species (Maranesi et al., 2021; Abumaghaid et al., 2022). Previous studies have shown that BDNF/NT-4-TrkB signaling is required for follicle assembly and growth, as blockade of both BDNF and NT-4 impairs follicular assembly and increases oocyte death (Ojeda et al., 2000; Spears et al., 2003). Recently, Ab4B19, a TrkB agonist antibody, was developed to treat premature ovarian failure associated with dysfunction of BDNF/TrkB signaling (Qin et al., 2022). Although these neurotrophic factors play a pivotal role in ovarian development, However, studies to date have focused mainly on the functions of only some neurotrophins (NGF, BDNF, and GDNF). The purpose of this study is to investigate the physiological and functional roles of NT-4 in porcine ovarian development.

Previous studies have demonstrated that the NT-4 mRNA and protein are expressed in granulosa cells, CCs, and oocytes (Dissen et al., 1995; Dissen et al., 2002; Seifer et al., 2006; Kim et al., 2021). In particular, many studies have indicated that NT-4 improves follicular assembly *in vitro* (Dissen et al., 1995; Ojeda et al., 2000; Anderson et al., 2002; Dissen et al., 2002; Harel et al., 2006; Farhi et al., 2011). However, it is still unknown whether NT-4/TrkB signaling is involved in the maturation and development of COCs in antral follicles as well as early follicular development. To determine whether NT-4-related signaling is involved in IVM of porcine oocytes, NT-4 and its receptors (TrkB and p75^NTR^) were detected and localized in *in vitro* matured COCs using immunofluorescence staining. In the CCs, both NT-4 and phospho-TrkB were observed strongly in the cytoplasm, and total TrkB and p75^NTR^ were generally distributed throughout the cytoplasm. Similarly strong patterns of NT-4 expression and phospho-TrkB were observed in matured oocytes. Total TrkB was mainly expressed on the surface of matured oocytes, whereas p75^NTR^ was distributed evenly throughout matured oocytes. These findings indicate that NT-4-related signaling pathways are involved in the maturation and development of porcine COCs.

The neurotrophin receptor p75^NTR^ plays essential roles in the development, survival, and death of neurons (Dechant and Barde, 2002). In ovarian cells, there are slight differences in the p75^NTR^ mRNA and protein expression depending on species. In mice, the *NGFR* mRNA is expressed in all follicular cells (mural granulosa cells and CCs), except oocytes (Kawamura et al., 2005); in cattle, the p75^NTR^ mRNA (Da Silva et al., 2005) and protein (Levanti et al., 2005) are expressed in both oocytes and CCs. In porcine studies, both p75^NTR^ mRNA (Lee et al., 2007; Kim et al., 2021) and protein (Levanti et al., 2005; Kim et al., 2021) have been detected in all follicular cells (granulosa cells, CCs, and oocytes). In our previous study, NT-4-related signaling pathways were identified in response to the MAPK/ERK pathway in matured porcine COCs by qPCR analysis (Kim et al., 2021). In the current study, the relative mRNA expression levels of *NGFR* and its downstream gene, *NFKB1*, were analyzed in matured porcine COCs upon NT-4 treatment during IVM to confirm the involvement of the NT-4/p75^NTR^-related signaling pathway. The results showed no significant differences in the *NGFR* mRNA expression levels in both matured COCs and oocytes between the control and NT-4 treatment groups. However, the mRNA expression levels of *NFKB1* were significantly higher (*p* < 0.05) in both NT-4-treated CCs and oocytes than in the control. In a previous study, NT-4 (100 ng/ml) was added during *in vitro* growth of mouse preantral follicles, and its effects were confirmed by single-cell RNA sequencing (Guo et al., 2021). Oocytes and granulosa cells were collected from preantral follicles on day 6 of NT-4 treatment; it was found that NT-4 initially regulated the transcriptional processes, primarily, but affected cell fate and development at the end of the *in vitro* growth. In addition, *NFKB1* was demonstrated to be a downstream effector of neurotrophin signaling. Another study has suggested that the neurotrophin/p75^NTR^ signaling pathway is mediated by the activation of NF-κB, which promotes cell survival (Karin and Lin, 2002). Collectively, the results of the present study show that the supplementation with NT-4 during IVM promotes the maturation of porcine oocytes and CCs by upregulating *NFKB1* transcription via the neurotrophin/p75^NTR^ signaling pathway.

This study also determined the relative expression levels of transcription factors in matured oocytes and demonstrated that NT-4 supplementation during IVM was beneficial for oocyte maturation and subsequent embryonic development. Both GDF9 and BMP15, which are representative oocyte secretory factors, are important for cyst breakdown, follicular development, and oocyte maturation (Elvin et al., 2000). In this study, NT-4 supplementation (10 ng/ml) during IVM significantly increased the mRNA expression levels of *GDF9* and *BMP15* in matured porcine oocytes, suggesting that NT-4 improves oocyte quality and participates in the secretion and paracrine interactions of GDF9 and BMP15 during porcine oocyte maturation. Furthermore, to confirm the effect of NT-4 supplementation during IVM on subsequent embryonic development of porcine oocytes after IVF, the relative mRNA expression levels of *CD9* were compared between NT-4-treated and control oocytes. In pigs, CD9 is mainly expressed in the plasma membrane of oocytes during early follicular development and meiotic maturation and is involved in sperm–oocyte interactions during fertilization (Li et al., 2004). The study showed a significant increase in the level of the CD9 protein between the germinal vesicle stage and the MII stage of porcine oocytes, in particular, during IVM. The present study confirmed that the expression level of the *CD9* transcript was significantly (*p* < 0.05) higher in NT-4-treated oocytes than in the control. NT-4 supplementation during IVM also significantly increased the monospermy rate, fertilization efficiency, and subsequent embryonic developmental potential after IVF. Thus, these findings indicate that NT-4 enhances fertility and oocyte maturation in pigs.

DNA methylation is an epigenetic mechanism and a critical process in oogenesis and early embryonic development (Uysal et al., 2015). DNA methylation is catalyzed by different types of DNA methyltransferases (DNMTs) involved in two different processes: maintenance and *de novo* methylation. Among the DNMTs, DNMT1 mainly functions in maintenance DNA methylation, while the functions of DNMT3A and DNMT3B are related to *de novo* DNA methylation (Vassena et al., 2005). In particular, the temporal mRNA expression of *DNMT3A* shows species-specific patterns during oocyte maturation and early embryonic development (Vassena et al., 2005). The *DNMT3A* mRNA expression has been previously reported in oocytes from mice (Kaneda et al., 2004; Vassena et al., 2005), cows (O'Doherty et al., 2012), monkeys (Vassena et al., 2005), humans (Huntriss et al., 2004), and pigs (Braga et al., 2019). According to a previous study, the *DNMT3A* transcription level significantly increased (*p* = 0.02) in the matured oocytes, compared with those in immature oocytes, of only prepubertal gilts (Braga et al., 2019). Similarly, *DNMT3A* was shown to be transcribed throughout oogenesis in rhesus monkeys (Vassena et al., 2005); however, the mRNA expression level increased significantly as oocytes matured from the germinal vesicle stage to the MII stage. Therefore, Braga et al. (Braga et al., 2019) suggested that the *DNMT3A* mRNA accumulated in matured porcine oocytes because they contained sufficient amounts of molecular transcription factors associated with the epigenetic mechanisms necessary for early embryonic development. The present study confirmed that *DNMT3A* mRNA expression significantly increased in NT-4-treated matured oocytes. Furthermore, when oocytes supplemented with NT-4 during IVM were evaluated for subsequent embryonic development after SCNT, significant increases were found in both the BL formation rate and total cell number in BLs. Although further studies are needed to determine how NT-4 regulates epigenetic molecular mechanisms during IVM, NT-4 supplementation leads to the upregulation of *DNMT3A* in matured porcine oocytes and enhances their subsequent embryonic capacity after SCNT.

Maturation promoting factor (MPF) is essential for the resumption of meiosis in oocytes (Murray et al., 1989). Previous studies have demonstrated that MPF activation induces phosphorylation of many protein kinases involved in the regulation of oocyte maturation in COCs through the MAPK signaling pathway including ERK1/2, c-Jun N-terminal Kinase 1/2, and p38 MAPK (Schmitt and Nebreda, 2002; Vigneron et al., 2004; Salhab et al., 2011; Chouzouris et al., 2017). In particular, activation of both MAPK and EGFR in pigs is required for the resumption of meiosis by gonadotrophins (Prochazka et al., 2003; Liang et al., 2005). Previous studies have shown that growth differentiation factor 8 activates p38 MAPK to improve porcine oocyte maturation (Yoon et al., 2017), and ganglioside GT1b activates EGFR-dependent ERK1/2 in porcine COCs to promote oocyte maturation and cumulus expansion (Kim et al., 2020). Therefore, as in previous studies, we confirmed whether NT-4 activates MAPK or EGFR in porcine COCs. In this study, supplementation of NT-4 during porcine IVM significantly (*p* < 0.05) increased the phosphorylation level of ERK1/2 in CCs. However, supplementation of NT-4 did not increase the levels of p38 MAPK and EGFR in CCs. As a result of confirming the ERK1/2 level of oocytes based on the results obtained from the CCs, it was confirmed that the supplementation of NT-4 significantly increased the total (*p* < 0.01) and phosphorylated (*p* < 0.05) ERK1/2 levels in oocytes. A previous study reported that EGF and BDNF promote metastasis and proliferation of ovarian cancer cells by transactivating TrkB and EGFR, respectively (Qiu et al., 2006). Another study has shown that EGF can transactivate the Trk receptor during early cortical development in newborns (Puehringer et al., 2013). Puehringer et al. (Puehringer et al., 2013) showed for the first time that TrkB and TrkC activation in neonatal early cortical neurons depend on transactivation by EGFR signaling, but not on BDNF and neurotrophin-3. In the present study, we did not investigate whether EGF and NT-4 transactivate EGFR and TrkB, respectively. However, we found that NT-4 supplementation in IVM medium significantly (*p* < 0.05) increased the level of ERK1/2 phosphorylation in the porcine COCs. Therefore, further analyses are needed on how NT-4 promotes phosphorylation of ERK1/2 in porcine COCs.

Cumulus expansion is essential to achieve successful meiotic maturation and developmental capacity of oocytes (Nevoral et al., 2015). Especially, ERK1/2 activation is important for the maturation of COCs (Su et al., 2002; Zhang et al., 2015; Ma et al., 2021). Our previous study reported that the addition of 10 ng/ml NT-4 helped the expansion of porcine CCs and upregulated cumulus expansion enabling factors (CEEFs) such as *HAS2* and *TNFAIP6* in the CCs (Kim et al., 2021). It is well known that various transcription factors including *HAS2* and *TNFAIP6* stimulate CC expansion, and the ERK1/2 activation by oocyte secretory factors promotes the resumption of meiosis and expansion of CCs (Su et al., 2002). Similary, previous studies have demonstrated that potential biomarkers of oocyte quality, such as GDF9 and BMP15, upregulate CEEFs and induce EGFR expression in CCs (Sasseville et al., 2010; Su et al., 2010; Arroyo et al., 2020). EGF-like peptides, such as amphiregulin, epiregulin, betacellulin, and neuregulins 1–4, which are triggered by the luteinizing hormone surge before ovulation, bind to EGFR on CCs and activate ERK1/2 (Vaccari et al., 2009; Su et al., 2010). Subsequently, phosphorylated ERK1/2 hyperphosphorylates the gap junction protein connexin 43, thereby disrupting gap junction communications between oocytes and CCs (Sela-Abramovich et al., 2005; Kidder and Vanderhyden, 2010). This signaling network is involved in oocyte maturation and cumulus expansion. Therefore, the EGFR/ERK pathway plays a very important role in mammalian oocyte growth and development (Hwang et al., 2018b; Abbassi et al., 2021; Alzahrani and Saadeldin, 2021). The current study showed no significant difference in the levels of total and phosphorylated EGFR in NT-4-treated CCs. Taken together, NT-4 upregulates the expression of *HAS2* and *TNFAIP6* in CCs (Kim et al., 2021) and stimulates the expression of *GDF9* and *BMP15* in oocytes, thereby helping the CC expansion and improving the quality of porcine oocytes. In addition, NT-4 regulates ERK1/2 in an EGFR-independent manner in porcine COCs and is involved in CC expansion and oocyte maturation. However, further studies of blocking ERK1/2 signaling with MEK inhibitors should be performed to investigate whether NT-4 enhances oocyte quality through activation of ERK1/2 in porcine COCs.

In conclusion, to the best of our knowledge, this study is the first to investigate the physiological roles of NT-4 in porcine ovarian development. The present study demonstrated the presence of NT-4, TrkB, and p75^NTR^ in matured porcine COCs, suggesting that NT-4-related signaling pathways are involved in the maturation and development of porcine COCs. NT-4 promoted the maturation of COCs by upregulating *NFKB1* transcript via the neurotrophin/p75^NTR^ signaling pathway and enhanced oocyte quality by upregulating *GDF9*, *BMP15*, *CD9*, and *DNMT3A* transcripts. The level of phosphorylated ERK1/2 was significantly higher in NT-4-treated CCs. Both total and phosphorylated ERK1/2 levels were significantly higher in NT-4-treated than in untreated oocytes. Furthermore, NT-4 improved subsequent embryonic development after IVF and SCNT. Collectively, NT-4 improves to oocyte maturation and CC expansion, and promotes ERK1/2 phosphorylation in porcine COCs during IVM.

## Data Availability

The original contributions presented in the study are included in the article/Supplementary Material, further inquiries can be directed to the corresponding author.

## Supplementary Material

Supplementary Figure 1 Protein expression levels of the epidermal growth factor receptor (EGFR) in porcine cumulus cells (CCs) after in vitro maturation (IVM). Western blots for total EGFR (T-EGFR), phosphorylated EGFR (P-EGFR), and GAPDH in CCs after IVM. The levels of T-EGFR **(B)**, P-EGFR **(C)**, and the ratio of P-EGFR to T-EGFR **(D)** in CCs **(A)**. The results were normalized to those of GAPDH. Values represent the mean ± SEM. The experiment was replicated four times.

Supplementary Figure 2 Protein expression levels of the p38 mitogen-activated protein kinase (p38 MAPK) in porcine cumulus cells (CCs) after in vitro maturation (IVM). Western blots of total p38 MAPK (T-p38 MAPK), phosphorylated p38 MAPK (P-p38 MAPK), and GAPDH in CCs after IVM. The levels of T-p38 MAPK **(B)**, P-p38 MAPK **(C)**, and the ratio of P-p38 MAPK to T-p38 MAPK **(A)** in CCs (a). The results were normalized to those of GAPDH. Values represent the mean ± SEM. The experiment was replicated three times.

Supplementary Table 1 Antibody lists for immunofluorescence analysis.

Supplementary Table 2 Primer lists for qPCR.

Supplementary Table 3 Antibody lists for western blotting.

Supplementary Table 4 Antibody lists for capillary western blotting.
