# Supplementary material for: Physiological and Functional Roles of Neurotrophin-4 During In Vitro Maturation of Porcine Cumulus–Oocyte Complexes
Source: Front Cell Dev Biol. 2022 Jul 8;10:908992. doi: 10.3389/fcell.2022.908992 (PMC9310091; doi:10.3389/fcell.2022.908992)
Supplement: Supplementary file 1 [file Table1.docx]

**Supplementary table 1. Antibody lists for immunofluorescence analysis.**

| **Antibody** | **Host** | **Dilution** | **Cat.** |
| --- | --- | --- | --- |
| **Anti-NT-4** | Mouse | 1:200 | NBP1-47897 |
| **Anti-TrkB** | Mouse | 1:200 | sc-377218 |
| **Anti-Phospho TrkB** | Rabbit | 1:200 | PA5-36695 |
| **Anti-p75^NTR^** | Mouse | 1:200 | 14-9400-82 |
| **Alexa Fluor 488** | Mouse or Rabbit | 1:400 | A11029 (Mouse), A11034 (Rabbit) |
| **Alexa Fluor 594** | Mouse | 1:400 | A11032 |

**Supplementary table 2. Primer lists for qPCR.**

| **mRNA** | **Primer sequences** | **Product size**  **(bp)** | **GenBank**  **accession number** |
| --- | --- | --- | --- |
| ***ZAR1*** | F: 5'-AAAAAGGCTCACTTGTCTGC-3' | 196 | XM_021100512 |
|  | R: 5'-CTCCTGCCCAGTAAAACTTC-3' |  |  |
| ***DPPA3*** | F: 5'-CTGAGTAGGTTGAGCCCACA-3' | 281 | XM_021093127 |
|  | R: 5'-CCAAAAGAGGCAAAACCTGA-3' |  |  |
| ***NPM2*** | F: 5'-GGAAGCAGGACTGTAAGCTC-3' | 199 | NM_021071936 |
|  | R: 5'-AACGTGACAGGAGGAGAAAG-3' |  |  |
| ***GDF9*** | F: 5'-GGTTCCAGCTTCATTCAATC-3' | 120 | NM_001001909 |
|  | R: 5'-ACAATCCAGTTGTCCCACTT-3' |  |  |
| ***BMP15*** | F: 5'-CCATCATCCAGAACCTTGTC-3' | 154 | NM_001005155 |
|  | R: 5'-CAGGACTGGGCAATCATATC-3' |  |  |
| ***CD9*** | F: 5'-AGACCAAGAGCATCTTCGAG-3' | 226 | NM_214006 |
|  | R: 5'-ACCTGATCCTTGTGGGAATA-3' |  |  |
| ***DNMT1*** | F: 5’-CCTCTATGGACGGCTTGAGT-3’ | 185 | NM_001032355 |
|  | R: 5’-GGTGCTTGTCCAGGATGTTG-3’ |  |  |
| ***DNMT3A*** | F: 5'-CAATAACCACGACCAGGAAT-3' | 220 | XM_021085534 |
|  | R: 5'-GTACATGATCTTGCCCTGGT-3' |  |  |
| ***DNMT3B*** | F: 5'-TAAGACTCGAAGACGCACAG-3' | 196 | XM_021077256 |
|  | R: 5'-ATTTTTCCGACCACAGGATA-3' |  |  |
| ***NGFR*** | F:5'-TGGAGATGGAGATGATATGGA-3' | 316 | NM_001244828 |
|  | R: 5'-GGCAATCTCCAATTAGAAGC-3' |  |  |
| ***NFKB1*** | F: 5'-CTACCAGACACCCTTGCACT-3' | 222 | NM_001048232 |
|  | R: 5'-ATAGCGTTCAGACCTTCACC-3' |  |  |
| ***RN18S*** | F: 5’-CGCGGTTCTATTTTGTTGGT-3’ | 219 | NR_046261 |
|  | R: 5’-GGTCATTTCCGACTGAAGAG-3’ |  |  |
| ***GAPDH*** | F: 5’-GTCGGTTGTGGATCTGACCT-3’ | 207 | NM_001206359 |
|  | R: 5’-TTGACGAAGTGGTCGTTGAG-3’ |  |  |

**Supplementary table 3. Antibody lists for western blotting.**

| **Antibody** | **Host** | **Dilution** | **Cat.** |
| --- | --- | --- | --- |
| **Anti-GAPDH** | Rabbit | 1:1000 | 2118L |
| **Anti-ERK1/2** | Rabbit | 1:1000 | 9102S |
| **Anti-phospho ERK1/2** | Rabbit | 1:1000 | 9101S |
| **Anti-EGFR** | Mouse | 1:400 | sc373746 |
| **Anti-phospho EGFR** | Rabbit | 1:400 | 44-788G |
| **Anti-p38 MAPK** | Rabbit | 1:700 | 9102S |
| **Anti-phospho p38 MAPK** | Rabbit | 1:700 | 9101S |
| **HRP-conjugated antibody** | Mouse  or Rabbit | 1:1000 (Mouse)  or 1:3000 (Rabbit) | 7076S (Mouse)  or 7074S (Rabbit) |

**Supplementary table 4. Antibody lists for capillary western blotting.**

| **Antibody** | **Host** | **Dilution** | **Cat.** |
| --- | --- | --- | --- |
| **Anti-GAPDH** | Rabbit | 1:50 | 2118L |
| **Anti-ERK1/2** | Rabbit | 1:50 | 9102S |
| **Anti-phospho ERK1/2** | Rabbit | 1:25 | 9101S |
| **Anti detection module** | Mouse or Rabbit | - | PSDM-002 (Mouse)  or PSDM-001 (Rabbit) |
