# Supplementary figures and images for: Physiological and Functional Roles of Neurotrophin-4 During In Vitro Maturation of Porcine Cumulus–Oocyte Complexes
Source: Front Cell Dev Biol. 2022 Jul 8;10:908992. doi: 10.3389/fcell.2022.908992 (PMC9310091; doi:10.3389/fcell.2022.908992)

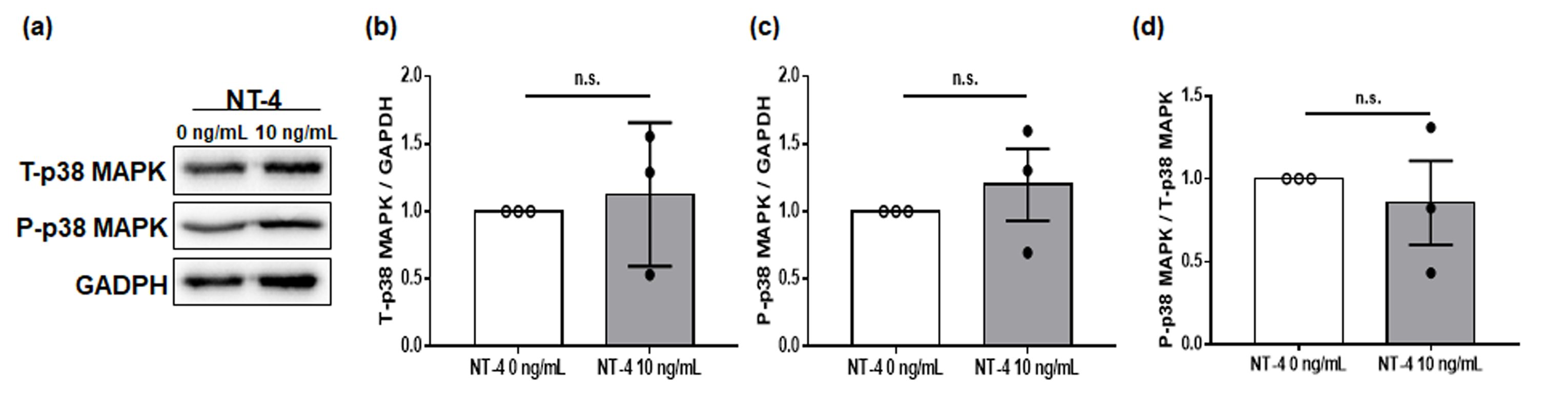

Supplement: Supplementary file 2 [file Image2.TIF]

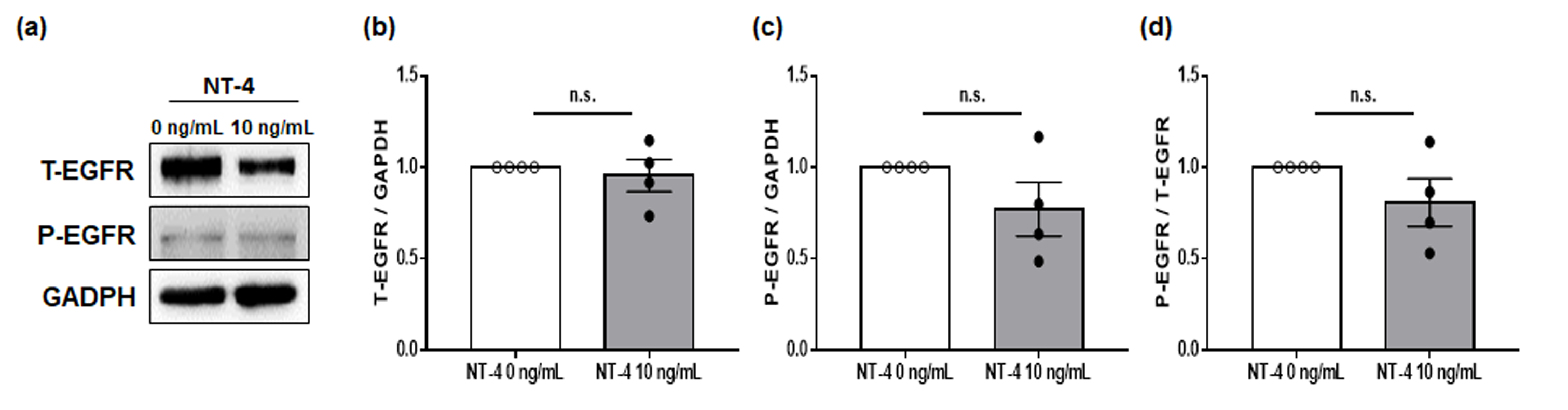

Supplement: Supplementary file 3 [file Image1.TIF]
